# Ethical considerations of artificial intelligence in veterinary medicine decision-making

**DOI:** 10.3389/fvets.2026.1780868

**Published:** 2026-02-13

**Authors:** Matt Heinlein

**Affiliations:** TLC Animal Hospital, El Paso, TX, United States

**Keywords:** accuracy, artificial intelligence, bias, decision- making, ethics, medicine, veterinary

## Abstract

The rapid growth and development of Artificial Intelligence (AI) is leading to a paradigm shift across multiple disciplines of decision-making. Veterinary medicine is an area wherein this proliferation offers profound potential for advancement, but is also ripe with potential ethical dilemmas resulting from the assimilation of AI technology into the decision-making process. While AI can increase accessibility of advanced veterinary care and improve efficiency of clinical and administrative workflow, the successful implementation of it into veterinary decision making requires assessment of key areas. These areas are the accuracy and reliability of AI diagnostic interpretations, the ethical implications of bias in AI algorithms, stewardship of privacy and personal data, and the balance of innovation with legal and professional responsibilities of animal welfare. Results of this review found that AI should aid, not replace, veterinary professional decision-making. To that end, continued research into accuracy and vigilance to mitigate bias is necessary, foundational standards for AI use and education must be enacted, and further research into the effect of AI on clinically ambiguous cases is imperative to safeguard the ethical standards of veterinary decision-making.

## Introduction

The proliferation of Artificial Intelligence (AI) technology is ingraining itself into an increasing number of daily activities in everyday life. The promise of AI has become a force for paradigm change, as algorithmic models reduce human involvement in decision-making and societal reliance on AI for evaluating information increases (1). Technology does not advance in a strictly linear manner. Roadblocks to technological advancement include areas such as accessibility, affordability, processing power, and speed. Increases in any of these areas will exponentially increase the likelihood and speed of the next advancement. It bears in mind asking whether the speed of AI advancement—and the rate of adoption—have circumvented traditional ethical frameworks, presenting new challenges for decision-making.

Veterinary medicine is a field that offers numerous opportunities to leverage AI in decision-making, and the rapid pace of technological advancement is leading to its ubiquitous integration into veterinary practice (2). AI applications are available for a variety of clinical and professional uses, such as client communication, scheduling, document generation, record-keeping, and diagnostic evaluation. The use of AI models is not limited to the clinical setting. Research and education are two other areas apt for the adoption of AI into their processes (3). A 2024 survey of 3,968 veterinary professionals found that 39.2% used AI in medical practice and 69.5% used AI for professional/administrative tasks (4).

While further integration of AI has the potential to drive positive change and advancements, the unique nature of veterinary medicine poses complex ethical considerations regarding the use of AI technologies for decision-making and support (5). The scope of this review is to evaluate existing AI technology in veterinary decision-making, discuss its potential for positive advancements, and assess its impact on ethical considerations within the field. Ethical dilemmas directly arising from the use of AI will be identified and detailed. The goal of this review is to identify gaps in the literature and identify areas that warrant recommendations for further research or regulatory consideration.

## Overview of AI applications in veterinary medicine: opportunities and considerations

An important distinction must be made to understand the current capabilities of AI technology. Narrow AI systems are designed to perform a single task or a limited set of tasks, with outputs based on problem-solving, pattern recognition, and reasoning guided by programmed parameters and preloaded datasets. General AI is a strictly theoretical concept in which advanced AI models would actively learn and apply knowledge to make independent decisions across potentially unlimited disciplines (3). As of the writing of this paper, general AI does not exist. All available AI systems today are a form of narrow AI, and their capabilities should be understood as such.

Sobkowich (3) details a wide range of commonly used AI applications in veterinary clinical workflows. Workflow automation includes such opportunities as client communication, medical scribes, appointment scheduling, and inventory management. The tools available to remove the human time cost of scheduling appointments or transcribing phone calls for training offer the promise of improved administrative efficiency. AI transcribed conversations with pet owners can be instantly uploaded to the patient record and streamline medical record maintenance. Automating inventory management can reduce the cost of goods sold by reducing shrinkage, and it can also prevent running out of necessary stock.

AI diagnostic tools can support clinical evaluation of various imaging modalities or cytological interpretation. Digital AI radiology services offer algorithmic screening of images, which have the potential to improve efficiency, accuracy, and consistency in the diagnostic interpretation and treatment planning of veterinarians (6). AI-powered point-of-care diagnostic tools provide cytological slide evaluation for areas such as hematology, fecal analysis, urine sediment analysis, and dermatology, thereby enhancing the capabilities of point-of-care instruments to more closely match those of a reference laboratory (7).

AI can be used in educational settings and practical skills training through in-clinic simulations and augmented reality. An example of this would be instructional overlays that provide real-time instruction and feedback to veterinary students on surgical rotations, or AI-curated educational platforms that can personalize learning support for individual students. Predictive analytics can also assist with early-detection systems and lab sample prioritization, as well as forecasting pathological trends in epidemiology (3).

The application of AI to these realms of veterinary decision-making stands to advance patient care, improve client service, and optimize clinical processes, while also offering new avenues to expand research, education, and animal welfare initiatives. However, the rapid advancement and general acceptance of AI create ethical concerns that must be addressed (8). This research found the questions of ethical concerns to be as follows:

1. Can veterinarians rely on the accuracy and reliability of AI-generated information for high-stakes decision-making?
2. What are the ethical implications of bias in AI datasets and algorithmic outputs?
3. How can veterinary professionals ensure ethical use of data and maintain stewardship of privacy and security?
4. How can veterinary professionals balance innovation with their legal and professional responsibilities assigned to patient care and animal welfare?

## Accuracy and reliability

There is no single way to build a diagnostic algorithm. However, a generally accepted sound practice is to train a convolutional neural network (CNN), with supporting learning models fine-tuning the process and, ideally, reducing errors. CNNs are advanced filter systems designed to recognize and identify increasingly precise aspects of images as they move down through finer layers of the filter system (7). For example, the first filter may recognize general shapes and outlines; the next level may identify image orientation; the third level may recognize body cavities; and this continues down to optimize image recognition through finer details, i.e., species, specific organs, or cellular structure, if using an AI cytology product. The images are then compared against a standard set of “normal” cases and “abnormal” examples of the pathology that the developers are training the AI to detect. Ideally, board-certified veterinary radiologists should be directly involved in developing and training AI models on standard image recognition datasets and image interpretation (6). The same should hold for cytology AI models, with board-certified pathologists directly involved in their development and training (7).

Many of the algorithms behind learning models in commercially available veterinary AI systems are proprietary information. As such, there is an inherent lack of transparency in the decision-making parameters of a given system, and veterinary practitioners may not know the internal factors influencing an AI program’s determination or proposed diagnosis (8). The opacity of proprietary algorithms creates questions of reliability and accuracy. AHAA and Digitail (4) surveyed veterinary professionals’ concerns about AI adoption, and reliability and accuracy were cited as the top concern by a majority of respondents (70.3%).

Pomerantz et al. (9) conducted an experiment to assess the ability of Vetology AI^®^, a commercially available AI radiology tool, to recognize pulmonary masses on thoracic radiographs. Accuracy, balanced accuracy, specificity, and sensitivity were tested by reading 56 sets of radiographic images using Vetology AI^®^. The 56 cases featured disease consistent with pulmonary nodules on radiograph, as confirmed by other diagnostic modalities, e.g., CT, cytology, or histopathology. A control group consisted of an additional 32 sets of normal radiographs.

Pomerantz et al. (9) found that the AI model correctly indicated the presence of pulmonary masses in 31 of 56 confirmed positive cases, and accurately read 30 of 32 controlled negative cases. The AI’s clinical interpretation was accurate 69.3% of the time, with a balanced accuracy of 74.6%. Specificity was 93.75%, and sensitivity dropped to 55.4%.

A study by Ndiaye et al. (10) compared the performance of another commercially available AI radiology software, SignalRAY^®^, with that of 11 board-certified veterinary radiologists. A sample group of 50 radiographic studies was randomly selected and anonymized from an institutional PACS system, consisting of 10 feline and 40 canine studies. Each study was both read by each radiologist and processed with the AI software. Results were coded as either normal or abnormal depending on the radiologists’ diagnostic report and the AI model’s classification of findings.

The results of the experiment found that the AI software’s overall accuracy was on par with that of the highest-performing radiologist and exceeded that of the median-performing radiologist. In both low- and high-ambiguity cases, the AI maintained a high level of accuracy. However, the AI was more specific but less sensitive compared to the interpretations of the images by the radiologists (10).

The high specificity-to-low sensitivity comparison observed by both Pomerantz et al. (9) and Ndiaye et al. (10) indicated that the versions of diagnostic AI models studied are better at recognizing negative results than positive results under the testing parameters. The systems performed better at recognizing normal images than at recognizing abnormal findings, indicating a greater propensity to rule out disease than to verify its presence (9). AI diagnostics products have the potential to increase the efficiency, availability, and accuracy of veterinary diagnostic decision-making; however, they should not be viewed as a replacement for veterinary clinical judgment, but rather as another tool in the veterinarian’s box to gain a greater understanding of a patient’s clinical presentation (11). As AI continues to advance, and existing models are trained on more data, continued research is required to assess the accuracy and reliability of their results. Furthermore, additional research is warranted to understand the propensity of AI results to influence veterinarians’ decisions in ambiguous cases or when the algorithm generates an erroneous recommendation.

## The effect of bias

An extenuating circumstance of the opaque nature of proprietary AI algorithms is the introduction of bias to the datasets. A substantial limitation of current studies on AI applications in veterinary medicine decision-making is bias arising from a limited data pool (10). For example, AI algorithms in pathology and radiology can only be trained with images available to developers. If particular species, conditions, or age groups are over-represented in either software development or in continually building the dataset at the end-user level, then results can be heavily skewed (12). An ethical question then arises: is it morally responsible for the veterinarian to make clinical recommendations based on those results (8)? Could an AI system trained mostly on canine and feline patients be expected to reliably produce clinical evaluations for underrepresented species, i.e., exotic companion animals or livestock? If species popular amongst urban clinics are fed into the algorithm at a higher rate than those of rural communities, which may have less access to veterinary care, would the AI output then be biased towards greater accuracy for conditions more common in urban breeds? As evidenced in human healthcare, ingrained bias can lead to unequal patient care across underrepresented groups (12). The same is true of veterinary medicine. Unseen bias can influence clinical outcomes, exacerbating disparities in access to care and, at a broader level, eroding public trust in veterinary clinical decisions (1). Addressing bias is an ethical obligation to mitigate unequal access to care and disparities in animal welfare.

## Data privacy and security

A common assumption about the nature of privacy is that it confers a right on a person to prevent unauthorized use of their information (13). While that is not all the concept of privacy entails, it is a suitable definition for the context of data collection in veterinary AI models. Data security and privacy were cited as the second most common concern regarding AI in the AHAA and Digitail survey (4) at 53.9%. Veterinary considerations include not only patients but also clients’ sensitive data collected during routine clinical practice. Substantial amounts of owner-identifying data are present in electronic medical records, and veterinary facilities must ensure the security of personal sensitive data (14).

The complex digital infrastructure required to maintain AI models and datasets relies on cloud storage, third-party vendors, and integrated software. This increases the risk of cyberattacks because the overall amount of breach points increases (13). Even anonymized data can be matched with other datasets to identify individuals in the event of a malicious breach (5). Data breaches can jeopardize client trust in a veterinary practice and severely disrupt practice operations, incurring catastrophic financial and reputational costs. Veterinary hospitals should have sufficient protection protocols in place to safeguard private information, such as cyberattack insurance and malware protection.

Malicious intent is not the sole consideration of data protection. The intentional release of information to developers or third-party vendors can be part of the AI workflow (14). Non-malicious use of the data could apply to algorithm training, marketing data aggregation, and additional software integration. This raises questions about informed consent, as clients may not fully understand how their data may be shared. Veterinary professionals should keep such questions in mind when reviewing terms of service for AI systems in their practice (14). Ethical stewardship of confidential information requires veterinarians to understand how data might be shared to inform clients of the possibilities adequately.

## Professional responsibility, animal welfare, and regulations

AI-assisted decision-making presents an exciting opportunity to leverage technology in improving veterinary standards of care. However, the rapid development and complexity of AI technology have outpaced the establishment of guidelines, regulations, and best practices (6). Veterinary patients, by nature, lack legal agency and the ability to communicate to inform their own care. Instead, this agency is assigned to both veterinary professionals and pet owners through the Veterinary-Client-Patient Relationship (VCPR). This lack of self-agency potentially exposes veterinary patients to a greater risk of harm from AI than patients in the human medical field (5). The VCPR is a construct designed to ensure that informed decisions are made in the best interests of the patient, as determined by the veterinarian and the client. In this role, veterinary professionals must advocate for their patients and allow clients an opportunity to advocate for their pets.

The ethical dilemma of informed decision-making is two-fold. First, veterinarians may not understand the complexity of AI algorithms and therefore cannot explain how an AI-generated report arrived at a specific diagnosis. Second, there is a lack of a regulatory framework that standardizes when veterinarians must inform clients about the use of AI tools. Ethically, veterinarians should strive for the utmost transparency when disclosing the use of AI systems to clients (6).

The use of AI poses challenges for assigning responsibility for erroneous diagnoses and clinical interpretations (5). If an AI-generated interpretation misses pathology or overdiagnoses a case, who is ultimately responsible? Is it the algorithm, the developers, or the veterinarian who is liable? The veterinarian is responsible for maintaining standards of care and assessing the information provided by diagnostic tools. The scope of veterinary responsibility is defined by a region’s veterinary practice act, which does not regulate specific tools but rather how they are used by a licensed veterinarian (14). For the successful deployment of AI in veterinary medicine decision-making, it is imperative that AI models support, rather than replace, practitioners’ clinical judgment (11). Regulatory bodies, such as state certifying boards, should be encouraged to establish rules on the scope of AI use in veterinary practice to protect veterinary professionals, clients, and animal welfare from potential hazards posed by AI misuse in veterinary decision-making (6).

Competency, training, and AI literacy will be integral as the field moves toward further integration with AI decision-making support. Veterinarians implementing AI models into their decision-making processes should seek out continuing education on AI to understand the performance of veterinary AI systems (6). A survey of professional veterinary students at the University of California-Davis found that 80% of respondents reported having slight or no knowledge of AI, and 59% expected to use AI tools in their practice. Moderate to extreme interest in the potential opportunities for AI in veterinary medicine was acknowledged by 79% of respondents, but only 37% reported learning about AI concepts in their curriculum (2). Given the role AI is likely to play in the future of veterinary medicine, it would behoove veterinary colleges to establish standardized parameters for AI development and utilization education in their programs (6). This will facilitate future veterinarians’ competence in AI applications, enabling them to critically evaluate the integration of AI into their practice (2). The potential benefits AI innovation offers to veterinary medicine must be continually scrutinized to balance technological advancement with monitoring of patient welfare.

## Discussion

Like many aspects of the world today, AI holds the potential for positive transformative power in veterinary medicine (6). However, AI should be used to support veterinary professionals’ clinical skills, but not to replace them. Continued research is necessary to assess the clinical performance of AI diagnostics, to increase the size of the tested data pool, and to monitor for signs of bias and inaccuracy. Governing and licensing bodies should set standards for the ethical use of AI in veterinary medicine and for safeguards for the collection and storage of personal data. Veterinary educational institutions must adapt to changes in the veterinary world that their students will experience and implement curricula that prepare veterinary professionals to understand and critically assess AI’s performance in their decision-making. This review found the need for further research into the use of AI in areas of ambiguous clinical evaluation. What is the propensity of AI to alter the clinical decision course of a veterinarian when patient presentation or clinical judgement disagrees with the AI-generated interpretation? Veterinary professionals have a professional duty and moral responsibility to safeguard the welfare of their patients and clients against the potential ethical risks of AI technological adoption.
